# Cognitive Interviewing during Pretesting of the Prefinal Afrikaans for the Western Cape Disabilities of the Arm, Shoulder and Hand Questionnaire following Translation and Cross-Cultural Adaptation

**DOI:** 10.1155/2020/3749575

**Published:** 2020-10-10

**Authors:** Susan de Klerk, Christina Jerosch-Herold, Helen Buchanan, Lana van Niekerk

**Affiliations:** ^1^Division of Occupational Therapy, Stellenbosch University, Cape Town 8000, South Africa; ^2^School of Health Sciences, University of East Anglia, Norwich NR4 7TJ, UK; ^3^Division of Occupational Therapy, University of Cape Town, Cape Town 7925, South Africa

## Abstract

When patient-reported measures are translated and cross-culturally adapted into any language, the process should conclude with cognitive interviewing during pretesting. This article reports on translation and cross-cultural adaptation of the Disabilities of the Arm, Shoulder and Hand (DASH) questionnaire into Afrikaans (for the Western Cape). This qualitative component of a clinical measurement, longitudinal study was aimed at the pretesting and cognitive interviewing of the prefinal Afrikaans (for the Western Cape) DASH questionnaire highlighting the iterative nature thereof. Twenty-two females and eight males with upper limb conditions were recruited to participate at public health care facilities in the Western Cape of South Africa. Cognitive interviews were conducted as a reparative approach with an iterative process through retrospective verbal probing during a debriefing session with 30 participants once they answered all 30 items of the translated DASH questionnaire. The sample included Afrikaans-speaking persons from low socioeconomic backgrounds, with low levels of education and employment (24 of 30 were unemployed). Pragmatic factors and measurement issues were addressed during the interviews. This study provides confirmation that both pragmatic factors and measurement issues need consideration in an iterative process as part of a reparative methodology towards improving patient-reported measures and ensuring strong content validity.

## 1. Introduction

The Disabilities of the Arm, Shoulder and Hand (DASH) questionnaire is a well-researched, evaluative and discriminative, region-specific, patient-reported outcome measure (PROM) used by many clinicians and researchers in the field of therapy for the upper limb [1]. The DASH measures symptoms and aspects of activity and participation according to the nine domains outlined in the International Classification of Functioning, Disability and Health (ICF) in patients with musculoskeletal conditions of the upper limb across 30 items rated on a five-point Likert scale [1, 2]. Scores are calculated in the prescribed manner towards an overall DASH symptom and disability score with scores closer to zero indicating less symptoms and disability and scores closer to 100 indicating greater symptoms and disability (http://www.dash.iwh.on.ca/). The DASH, developed in 1996 by the Institute of Work and Health (IWH) in the context of Canada, has been translated into many languages from around the world with 12 language versions for developing countries [3]. Translation and cross-cultural adaptation are an accepted important component in ensuring PROMs, such as the DASH, continue to measure what they intend to measure following translation [4–6]. The process of translation and cross-cultural adaptation of the DASH is clearly outlined by the IWH and involves a five-step process that concludes with the final step of pretesting and cognitive interviewing reported on here [6]. The first four steps were completed and published elsewhere [7]. The process commenced with communicating the intent to translate into a new language version to the IWH, followed by forward translation of the DASH from English into Afrikaans done by two translators in step one. This was followed by synthesis of the translation during step two. Step three involved back translation of the synthesised new language version into English by two new translators. A prefinal version was created by an expert review committee (harmonisation) during step four [7].

The translation considered during the present study is the Afrikaans for the Western Cape DASH. The variation that exists in the Afrikaans language is often a product of social, cultural, situational, or geographical differences between people [8]. Afrikaans is spoken by 13.5% of the population of South Africa and most widely used in the Western and Northern Cape Province [9]. Variations however exist. The target population comprised Afrikaans-speaking persons of whom the majority are public health service users within the Western Cape of South Africa. The Afrikaans spoken by this population is characterised by dialectical use of language and code switching between nonstandard Afrikaans and English [8, 10, 11]. In the present study, shared decision-making (SDM) and community translation (CT) approaches were applied during the harmonisation (step four) of the DASH to Afrikaans for the Western Cape [7]. Harmonisation produced a prefinal version of the DASH, for pretesting and cognitive interviewing (CI).

Even though CI is undertaken by most researchers, as is evident from the many language versions that exist for this measure, publications routinely do not include details on the steps, methodology, or analysis. CI is a methodology developed in the 1980s to assist in understanding the cognitive processes involved in answering questionnaires in order to improve the design of the questionnaire, minimize response error, and improve content validity [12, 13]. The COnsensus-based Standards for the selection of health Measurement INstruments (COSMIN) group outlines CI as a methodology to evaluate the most important measurement property of PROMs, namely, content validity [14]. This approach is widely used during the translation and cross-cultural adaptation of the DASH in developing countries [3, 15–19] and advocated by experts in the field of translation and cross-cultural adaptation of PROMs [4, 5]. *Think aloud interviewing* and *verbal probing techniques* are the two main subtypes of CI [12, 13, 20]. Think aloud interviewing asks the participant to “think aloud” as they answer each item on the questionnaire. The interviewer documents the thoughts and/or thought processes that lead the participant to answer the question. Verbal probing techniques involve the use of cognitive probes once the participant has answered the question. The basic categories of probes include comprehension/interpretation, paraphrasing, confidence judgement, recall, specificity, and general [12, 13]. Advantages of verbal probing include control of the interview and ease of training of the participant. Lee reports specific issues during cross-cultural CI, including but not limited to procedural and practical considerations (pragmatic factors) and measurement issues such as usability, readability, accessibility, applicability, and relevance of the measure [21]. These issues can be compared with the COSMIN consideration of good content validity in PROMs, namely, relevance, comprehensiveness, and comprehensibility of the translated measure [14]. All require attention when making decisions during CI and are highlighted in the discussion. The steps, methodology, or analysis of cognitive interviews following translation and cross-cultural adaptation of the DASH have been documented to a very limited extent to date. The purpose of this study was therefore to conduct and report the iterative process of CI employed in pretesting the prefinal version of the Afrikaans for the Western Cape Disabilities of the Arm, Shoulder and Hand questionnaire (DASH-PAV), version 1.

## 2. Materials and Methods

### 2.1. Study Design

Qualitative study as a component of a clinical measurement longitudinal study aimed at the translation and cross-cultural adaptation of the DASH questionnaire to Afrikaans (for the Western Cape).

### 2.2. Research Context: Population and Setting

The research context is that of public health care facilities (tertiary and/or primary level health care facilities) in suburbs within the geographical area referred to as the Cape Flats in the Cape Metropole of the Western Cape (South Africa). Tertiary level public health care facilities offer specialist hand surgery and hand therapy services. Following specialist intervention, patients are referred to public sector primary level health care institutions where rehabilitation is offered to community members with a range of conditions including upper limb (UL) conditions. The population was from three suburbs (Bishop Lavis, Elsies Rivier, and Matroosfontein) which were established under *apartheid* [22]. Some characteristics of the population and setting can be seen in Table 1.

### 2.3. Sampling and Recruitment

Patients with any hand injury or condition were conveniently sampled for inclusion as per the criteria in Table 2. The study was approved by Stellenbosch University Health Research Ethics Committee (# S18/03/063) and the University of Cape Town Human Research Ethics Committee (744/2018). Permission was granted by the Western Cape Department of Health (WC_201806_029) to conduct the study at the proposed sites. Written consent was sought prior to participation.

Participants were either seen at tertiary or primary public sector health care facilities within the Cape Metropole. Patients were recruited after they arrived at the health care setting to be seen by an occupational therapist for a hand injury or condition. In the first round, 17 persons were recruited of which 15 met the inclusion criteria.

### 2.4. Data Collection: Cognitive Interviews

The IWH recommends pretesting which includes interviewing between 30 and 40 participants from the target population [6]. The document however does not refer to the interviews as CI specifically, but encourages the researcher to probe towards coming to an understanding of what was meant by each question, after participants complete the entire questionnaire [6]. This is in line with the verbal probing CI technique Willis described, which was applied in the current study [12, 20]. Therefore, CIs were conducted as a reparative approach with an iterative process through retrospective verbal probing during a debriefing session once participants answered all 30 items of the DASH-PAV (see Supplementary Material 1 for examples of verbal probes during CI). CI took place at Tygerberg Hospital, the Bishop Lavis Rehabilitation Centre, and Elsies Rivier Day Hospital between 14 August 2018 and 15 January 2019 in two rounds. The first author (principal investigator) initially interviewed 15 participants (1^st^ round), analysed the findings, revised the questionnaire, and commenced a second round of interviewing 15 participants. The first round of CIs was conducted between 14 and 23 August 2018 by the principal investigator (PI). In the second round, 15 persons meeting the inclusion criteria were recruited between 18 September 2018 and 15 January 2019.

### 2.5. Instrumentation and Data Management

Sociodemographic information (including age, gender, educational level, and employment status) in addition to information about the nature of the hand injury or condition were obtained from the participants and recorded on a data capture sheet. Participants were asked to complete all 30 items of the DASH-PAV, and the completion time was recorded. Verbal probing commenced, CIs were recorded on a smartphone, and the PI made notes during interviewing. The completed questionnaires and the recordings were stored safely by the PI. Refreshments and a pen to take home were offered as a token of appreciation for the extra time spent at the health care facility.

### 2.6. Data Analyses

**Figure 1:**
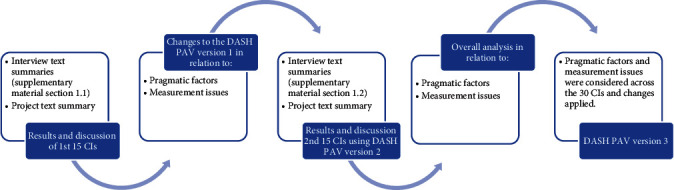
Iterative process followed during cognitive interviewing.

The text summary model was applied in the analysis of the data following CI [20]. Text summary is understood to describe dominant themes, conclusions, and problems as supported by the data [20]. Willis distinguishes between the *interviewer text summary* and the *project text summary* [20]. The interviewer text summary refers to the notes from one cognitive interview, and the project text summary refers to the compilation of all interviews within one project [20]. Project text summaries are provided in text for pragmatic factors and the measurement properties of usability, readability, accessibility, applicability, and relevance of the measure [21]. Interviewer text summaries of the first and subsequent round of CIs are presented considering the *target item*, *text summary*, and *recommendations*. Recommendations were implemented as revisions and tested in subsequent interviews. The iterative process of this reparative investigation that lead to the DASH-PAV version 3 is illustrated in Figure 1.

## 3. Results and Discussion

### 3.1. Cognitive Interview Demographics

During the first round of cognitive interviews, three men and 12 women were recruited and during the second round five men and 10 women across the three health care facilities. Demographic information is captured in Table 3.

CI results are presented and discussed in *project text summaries* relating to pragmatic factors and measurement issues in the section below. *Interview text summaries* (results and discussion) are presented for the first (section 1.1) and second round (section 1.2) of 15 CIs in the Supplementary Material 2.

### 3.2. Project Text Summary First Round of 15 CIs: Pragmatic Factors

Participants had basic literacy skills with low levels of education and were mostly unemployed indicating low socioeconomic status (Table 3). Four of the 15 participants in the first round of CIs did not bring their reading glasses to the occupational therapy appointment, as they did not expect to need to read anything. Another two participants struggled to read the text, but reported that they either did not have the financial means to have their eyes tested or they were awaiting an appointment to have their eyes tested. The interviews were conducted in the very small and overcrowded space within the therapy settings (often shared between the occupational therapy and physiotherapy service) characteristic of primary level health care in South Africa. Noise levels were high and privacy a challenge. However, recordings from the interview were clear (done with a smartphone), and in all instances, the interviewer and the interviewee were able to communicate and interact despite the less than ideal interview settings. Many participants commented that they hardly fill in forms anymore; however, two female participants with school age children reported completing forms for school. Participants were very grateful for the gifting of the pen after completing the questionnaire. Participant 2 (a 38-year-old male) said he was going to give it to his child for school as she did not have a pen. Incidents like this highlight the characteristics of the participants which Lee proposes as practical considerations when conducting cross-cultural CIs [21]. In the present study, there were marked socioeconomic, educational, and cultural differences between the interviewer (PI) and the participants of the study, which can lead to lack of openness on the part of the participants [21]. As per Lee's recommendation towards addressing some of the pragmatic factors and despite the challenges these add, all interviews were conducted in the “local sites” ([21], p.237). The PI adapted to participants not being able to read without reading glasses by purchasing three nonprescription reading glasses of various strengths (+2, +2.5, and+3) early in the first round of interviews, for participants to use if needed. In addition, despite the stated differences between the interviewer and interviewees, familiarity with the health care settings and 20 years' experience engaging with patients within these settings as well as the match between the target language and the mother tongue of the PI [21] assisted in mitigating some of these pragmatic factors.

### 3.3. Project Text Summary First Round of 15 CIs: Measurement Issues

The DASH was translated and cross-culturally adapted with the consideration of the practice of CT [10]. Through the process of CT, the receiver of the translation sets the norm for the translations, and therefore, it is important for the interviewer to comment on measurement aspects of usability, readability, accessibility, applicability, and relevance of the translation as described by Lee [21]. Readability refers to whether the terms used in the translated questionnaire are clear to participants; accessibility is concerned with whether the respondents understand the question and are able to provide a response; usability refers to the format and the ease of use, and applicability asks if the terms and concepts apply to the local context [21]. This relates to some of the ten criteria for content validity introduced by the COSMIN group, specifically aspects of relevance of the PROM (for the target population and context of interest) and comprehensibility (are the instructions, items, and response options of the PROM understood by the target population?) [14].

In the first round of 15 interviews, there were several problems with the measurement properties described above. Task performance of completing the questionnaire was a challenge as participants did not know how to proceed, even after reading the instructions (readability and accessibility). They also found the font size very small (usability). Some experienced memory and/or retrieval problems when having to recall the meaning of numbers 1 to 5 as an answer choice (usability and applicability). For example, in items 1 to 21, 1 represents *no difficulty*, 2 represents *mild difficulty*, 3 represents *moderate difficulty*, 4 represents *severe difficulty*, and 5 represents *unable*; participants who could not remember or recall these response choices had to look back up to column header, which caused them to lose their place, resulting in endorsing more than one response option in the same line. Most of the difficulties experienced during the first round of CIs were addressed in the interview text summaries (Supplementary Material, section 1.1 (available here)). The overall recommendation following the first round of 15 CIs to address the usability, readability, applicability, and accessibility (and in effect relevance and comprehensibility) included:

1. changing the font size to a slightly larger font
2. replacing the numbers (1–5) in all items with the response choices (words), as it applied to the specific test items. As a result, all references to circling the correct number in the instructions were changed to circling the correct answer in all instances

### 3.4. Project Text Summary Second Round of 15 CIs: Pragmatic Factors

The pragmatic factors experienced during the first round of CIs remained during the second round of interviews. Four participants used the reading glasses provided by the PI. Education levels and socioeconomic status of participants were similar to those in the first round of CIs. The ease of the interviewing did however improve, as a result of the PI gaining experience [12] in addition to changes made to the questionnaire following the first round of CIs. Persons were able to complete the questionnaire faster (6 participants took less than 8 minutes to complete the questionnaire), with the exception of a 33-year-old male who took 27 minutes. This interview was conducted in the very busy Elsies Rivier Day Hospital rehabilitation setting on a particularly busy day. The small space was overcrowded with patients and therapists. In addition, the participant had a grade 5 level of education and reported that he hardly reads. As a result, this interview was conducted more in accordance with the *think aloud* technique rather than by *verbal probing*, as the participant spontaneously started reading the questionnaire items out loud and reasoned his answer in a discussion with the PI [12]. Despite the length of time taken to complete the questionnaire, it was evident that he understood all the questionnaire items and was able to accurately respond in all instances. This interview highlighted that, firstly, the pragmatic factors as experienced during the CIs are characteristic of the health care settings within this context and will remain so in using the questionnaire in routine practice and, secondly, that these factors can be overcome towards improved clinical utility of the DASH.

### 3.5. Project Text Summary Second Round of 15 CIs: Measurement Issues

The measurement issues experienced during the first round of CIs were mostly addressed as a result of the changes made to the questionnaire before the second round CIs commenced (Supplementary Material, section 1.2 (available here)). Participants understood the instructions better and were in all instances able to complete the questionnaire without response errors such as marking two response options in one line. A recommendation was made in the text summary of the second round of CIs with regard to changing item 20 (manage transport needs (getting from one place to another)) to improve readability and usability of this item (see Supplementary Material, section 1.2 (available here)). Similar to the participants in the first round of CIs, the second round of participants reported the questionnaire to be written in an understandable language, accessible to them. Willis suggests that the researcher decides when to break out of the iterative process and when enough testing has been done [20]. This approach ensures that the “*best efforts toward analysis*” is not applied at the end of the CI process, but rather throughout the process, which complements the reparative methodology employed in the current study (refer to Figure 1) ([20], p. 53). The question of the appropriate number of CIs remains unclear. Willis suggests an iterative process where 8 to 12 interviews are conducted, findings are assessed, and preliminary analysis is done towards revising the test items, referred to as reparative investigation [12, 20]. This is then followed by another round of testing and CIs. The International Society for Pharmacoeconomics and Outcomes Research (ISPOR) task force for translation and cultural adaptation suggests that cognitive debriefing be conducted on a sample of 5 to 8 participants from the target population [5]. This however has to be considered against the IWH guidelines for CIs following translation of the DASH, which states that between 30 and 40 CIs have to be conducted [6]. In the present study, it was evident (after as little as 5 CIs) that few new measurement issues arose during the second round of CIs; the PI however continued in accordance with the IWH guidelines. Continuing to reach a target sample size of 15 during the second round of CI did serve as confirmation that changes made to the questionnaire were appropriate and applicable; however, when considering the reparative methodology, the current study could have been stopped following the first 5 CIs in the second round. The process reported above concluded the final version of the Afrikaans for the Western Cape DASH, to be further evaluated for structural validity.

### 3.6. Strength and Limitations

The iterative nature of the process is considered a strength. A limitation of this study is that the PI had no prior experience with CIs.

## 4. Conclusions

This study highlights the process of CI employed in the pretesting and cognitive interviewing of the prefinal version of the Afrikaans for the Western Cape DASH-PAV. It is recommended that pragmatic factors experienced during CIs be addressed with adaptations such as the availability of reading glasses or through experience with the target population and/or similarities between the interviewer and the target population. In addition, measurement issues have to be addressed and evaluated in subsequent interviews. A further recommendation is to consider stopping CIs once it is evident that pragmatic and measurement issues were addressed as part of a reparative methodology with an iterative approach following translation and cross-cultural adaptation of the DASH. This study also provides evidence for content validity of the newly translated and cross-culturally adapted Afrikaans for the Western Cape DASH questionnaire.

## Figures and Tables

**Table 1:** Population and setting characteristics.

Suburb	Bishop Lavis	Elsies Rivier	Matroosfontein
Area	2.58 km^2^	5.52 km^2^	25.44 km^2^
Population (per km^2^)	26,482 (10,247.48 per km^2^)	42,479 (7,701.39 per km^2^)	77,121 (3,031.69 per km^2^)
Households (per km^2^)	5,788 (2,239.73 per km^2^)	9,207 (1,669.22 per km^2^)	16,034 (630.31 per km^2^)
Percentage Afrikaans-speaking individuals	86.45%	77.45%	81.62%

km^2^ = square kilometre.

**Table 2:** Inclusion criteria.

Sample	30 participants
Inclusion criteria	Afrikaans-speaking persons, 18 years or older, with musculoskeletal hand injury or condition
	Seen at government health care facilities within the Western Cape of South Africa, situated in/or serving previously disadvantaged and low socioeconomic status populations within the Western Cape such as Bishop Lavis, Elsies Rivier, or Matroosfontein
	Basic literacy skills (ability to read the DASH-PAV or any other reading material)

**Table 3:** Patient demographics.

	1^st^ round CI (*n* = 15)	2^nd^ round CI (*n* = 15)
Ratio, female : male	12 : 3	10 : 5
Median age (min–max)	50 (38–83)	49 (23–66)
Median education level (min–max)^∗^	Grd 8 (Grd 1–Grd 12)	Grd 9 (Grd 5–Grd 12)
Ratio, employment to unemployment	3 : 12	3 : 12
Unemployed 60 yrs and older	2	1
Ratio, dominance right to left	13 : 2	14 : 1
Affected side		
Right	6	8
Left	5	5
Both right & left	4	2
Diagnosis		
Carpal tunnel syndrome	6	3
Distal radius fracture	1	1
Distal ulna fracture	1	0
Humerus fracture	1	0
Olecranon fracture	0	1
Extensor tendon injury	1	1
Flexor tendon injury	0	1
Fractured phalanx or metacarpal	1	1
Shoulder pain	3	1
Dislocation of IPJ	1	0
Finger amputations	0	1
RA of the wrist	0	2
Burns of the hands	0	1
Trigger finger	0	1
Trapeziectomy	0	1
	Median (min–max)	Median (min–max)
Duration of symptoms (months)	6 (0.4–72)	5 (1–72)
Time taken to complete (minutes)	9.1 (6.5–13.0)	8.3 (3.5–27.4)

^∗^Education level is described in terms of levels of education within the South African Education System (National Department of Basic Education) [23]. Grade 12 is the High School Diploma or National Senior Certificate (NSC) and is the main school-leaving certificate in South Africa. Min = minimum; Max = maximum; Grd = grade; IPJ = interphalangeal joint; RA = rheumatoid arthritis.

## Data Availability

The cognitive interview data used to support the findings of this study are available from the corresponding author upon request.

## Supplementary Materials

Supplementary 1 Cognitive interviewing probes.

Supplementary 2 The supplementary material file contains the interview text summaries of the 30 CIs.
