# Supplementary material for: Cognitive Interviewing during Pretesting of the Prefinal Afrikaans for the Western Cape Disabilities of the Arm, Shoulder and Hand Questionnaire following Translation and Cross-Cultural Adaptation
Source: Occup Ther Int. 2020 Oct 10;2020:3749575. doi: 10.1155/2020/3749575 (PMC7576342; doi:10.1155/2020/3749575)
Supplement: Supplementary 1 — Cognitive interviewing probes. [file 3749575.f1.pdf]

**Cognitive interviewing probes**

**Examples of verbal probes during cognitive interviewing**:

| **Cognitive probe** | **Example** | **Document during CI** |
|---|---|---|
| **Comprehension / Interpretation probe** | What does the term "bietjie moelik" mean to you?<br>What does the term…… mean to you?<br>Was the instructions clear?<br>What do you think of the name of the questionnaire?<br>What do you think about the layout of the questionnaire?<br>Is the font large enough? | |
| **Paraphrasing** | Can you repeat the question in your own words | |
| **Confidence judgement** | How sure are you that you are unable to do heavy household chores? | |

| | | |
|---|---|---|
| **Recall probe** | How do you remember you pain levels this past week? | |
| **Specific probe** | Why do you think you are “minder werd” due to your hand problem or injury? | |
| **General probe** | How did you get to your specific answer? Was it easy or hard to answer? | |
