# Supplementary material for: Cognitive Interviewing during Pretesting of the Prefinal Afrikaans for the Western Cape Disabilities of the Arm, Shoulder and Hand Questionnaire following Translation and Cross-Cultural Adaptation
Source: Occup Ther Int. 2020 Oct 10;2020:3749575. doi: 10.1155/2020/3749575 (PMC7576342; doi:10.1155/2020/3749575)
Supplement: Supplementary 2 — The supplementary material file contains the interview text summaries of the 30 CIs. [file 3749575.f2.pdf]

1.1. Interview text summary 1st 15 cognitive interviews

Target Item: Instruction to items 1 to 21: *Trek asseblief 'n sirkel om die nommer wat die beste by jou pas. Dink asseblief terug oor hoe dit vir jou die laaste week was wanneer jy antwoord. Hoe maklik of hoe moeilik was die aktiwiteit vir jou?*

[Draw a circle around the number that fits with you best. Think back about how it was for you in the last week when you answer. How easy or difficult was the activity for you?]

Text summary: Three of the 15 participants had injuries to their non-dominant hand. All three commented on the fact that they will not use their injured hand for doing the activities listed (especially the first 5 items, typically completed with the dominant hand) and were therefore unsure as to how to answer. Participant 15 (a 50 year male) shifted around in his chair, looked up and reported the following: "*Maar ek doen mos nie hierrie goed met my seer hand nie"* [But I don't do these things with my sore hand] The instruction sheet (first page) of the questionnaire states that they have to answer the question based on their ability irrespective of which hand is used in the execution of the task; they however did not follow this instruction when confronted with the instruction located directly above the set of items (item 1 to 21) on the second page of the questionnaire.

Recommendations: Rephrase to say: *Trek asseblief 'n sirkel om die ~~nommer~~* antwoord wat die beste by jou pas. Dink asseblief terug oor hoe dit vir jou die laaste week was wanneer jy antwoord. Hoe maklik of hoe moeilik was die aktiwiteit vir jou? Dit maak nie saak watter arm of hand jy gebruik om dit te doen nie.*

[Draw a circle around the ~~number~~* answer that fits with you best. Think back about how it was for you in the last week when you answer. How easy or difficult was the activity for you? It does not matter what arm or hand you use to do it.]

This repeats the instruction from the first page to ensure that they answer irrespective of which hand is used to do the activity.

*See Project summary: Measurement issues, point 2 in the body of the article

---

Target item: Item 7: *Om baie harde huiswerk te doen (soos venters was, wasgoed uitdroog of meubels skuif.*

[To do hard household tasks (like washing windows, to dry out washing or to move furniture]

Text summary: During initial general verbal probing regarding this item i.e. *How did you get to your specific answer?*, most participants used the word *swaar* [heavy] to describe the level of difficulty of the household tasks listed in the test item. Participant 5 (a 50 year old female)

reported: "*Dis swaar om dié goed te doen."* [It is heavy to do these things]. In subsequent interviews the researcher made use of paraphrasing probing by asking participants to repeat the question in their own words. Five of the seven participants probed in this manner used the word *swaar* [heavy] in rephrasing the question in their own words.
**Recommendations:** Replace the word *harde* [hard] with *swaar* [heavy].
**Target Item**: Item 21: *Om intiem te wees*<br><br>[To be intimate]
**Text summary**: In the source version of the DASH this item is: Sexual activities. During the harmonisation meeting this item was translated to *Om intiem te wees* [to be intimate] in order to reflect aspects of intimacy beyond sexual activities, as impacted by an arm, shoulder of hand injury or condition. This item was discussed with each of the 15 participants during the first stage of pre-testing. Five of the 15 participants did not know what *om intiem te wees* [to be intimate] means. Participant 6 (a 43 year old male) asked the meaning of the item while completing the questionnaire. After offering the meaning he responded by saying: "*Ooooo daai… baie mense sal nie vir hom verstaan nie"* [Oh, that. Lots of people will not understand that]. In asking him to paraphrase he responded by saying: "*Om seksueel intiem te wees"* [To be sexually intimate]. Participant 14 (a 58 year old female) understood the reference to intimacy but voiced that "*onse mense*" [our people] will not know the meaning of the word *intiem* [Intimate]. During comprehension / interpretation probing of this item three of 15 participants reported aspects of intimacy with their family or friends to be affected and did not equate the item to include reference to sexual activities only. Participant 1 (a 45 year old female) said: *"Om intiem te wees is uhm miskien byvoorbeeld om die kind 'n drukkie te gee en so aan te is is is… Want ek is nou bang…Hulle is ook self bang hulle maak my seer".* [To be intimate is maybe for example to give the child a hug and so on… Because now I am afraid… They are also afraid to hurt me]
**Recommendation**: Rephrase to: *Seksuele aktiwiteite* [Sexual activities] as per the source version for the second round of testing.
**Target Item**: Instruction to items 24 to 28: *Hoe erg was jou simptome die laaste week? Trek 'n sirkel om jou antwoord. [GEEN / BIETJIE / REDELIK / ERG / BAIE ERG]*<br><br>[How bad were you symptoms during the last week. Draw a circle around your answer. NONE / A LITTLE / SOME / BAD / VERY BAD]

**Text summary**: Six of 15 participants within the first round of interviews misinterpreted this instruction to be a question unrelated to the items to follow (item 24 to 28) and circled the response that best answered the question from their understanding, instead of the numeric response that follow in response to items 24 to 28.
**Recommendations**: Rephrase to say: *Hoe erg was die simptome hier onder in vraag 24 tot 28 die laaste week? Trek 'n sirkel om jou antwoord.* [How bad were you symptoms under in question 24 to 28 during the last week. Draw a circle around your answer]

1.2. Interview text summary 2nd 15 cognitive interviews

**Target Item**: Item 20: Om jou eie motor of public transport te gebruik<br>[To use your own vehicle or to use public transport]
**Text summary:** Five of 15 participants during the second round of CI's asked what they should answer as they do not have their own vehicle. In probing them to read the question again in full they would respond by saying that they do use public transport and then proceed in answering the question. As a result of the low socio-economic status of the participants in this study, most don't own their own vehicles, but routinely make use of public transport.
**Recommendation**: Change the test item to lead with the use of public transport followed by the use of your own vehicle: *Om public transport of jou eie motor te gebruik.* [To use public transport or your own vehicle]
